# Comparison of Validity and Reliability of Manual Consensus Grading vs. Automated AI Grading for Diabetic Retinopathy Screening in Oslo, Norway: A Cross-Sectional Pilot Study

**DOI:** 10.3390/jcm14134810

**Published:** 2025-07-07

**Authors:** Mia Karabeg, Goran Petrovski, Katrine Holen, Ellen Steffenssen Sauesund, Dag Sigurd Fosmark, Greg Russell, Maja Gran Erke, Vallo Volke, Vidas Raudonis, Rasa Verkauskiene, Jelizaveta Sokolovska, Morten Carstens Moe, Inga-Britt Kjellevold Haugen, Beata Eva Petrovski

**Affiliations:** 1 Center for Eye Research and Innovative Diagnostics, Department of Ophthalmology, Institute for Clinical Medicine, University of Oslo, Kirkeveien 166, 0450 Oslo, Norway; mia.karabeg@studmed.uio.no (M.K.); goran.petrovski@medisin.uio.no (G.P.); m.c.moe@medisin.uio.no (M.C.M.); 2 Department of Ophthalmology, Oslo University Hospital, Kirkeveien 166, 0450 Oslo, Norway; katrine08@live.no (K.H.); ellen.s.sauesund@gmail.com (E.S.S.); doktordag@icloud.com (D.S.F.); majaerke@gmail.com (M.G.E.); 3 Department of Ophthalmology, University Hospital Centre, University of Split School of Medicine, 21000 Split, Croatia; 4 UKLONetwork, University St. Kliment Ohridski-Bitola, 7000 Bitola, North Macedonia; 5 Clinical Development, Eyenuk Inc., Woodland Hills, CA 91367, USA; greg@eyenuk.com; 6 Faculty of Medicine, Tartu University, 50411 Tartu, Estonia; vallo.volke@gmail.com; 7 Automation Department, Kaunas University of Technology, 51368 Kaunas, Lithuania; vidas.raudonis@ktu.lt; 8 Institute of Endocrinology, Lithuanian University of Health Sciences, 50161 Kaunas, Lithuania; rasa.verkauskiene@gmail.com; 9 Faculty of Medicine, University of Latvia, Jelgavas Street 3, LV1004 Riga, Latvia; sokolovska.jelizaveta@gmail.com; 10 Norwegian Association of the Blind and Partially Sighted, 0354 Oslo, Norway; inga-britt.haugen@blindeforbundet.no

**Keywords:** diabetic retinopathy, artificial intelligence (AI), automated grading, EyeArt, diabetic macular edema, fundus photography, screening program, manual consensus grading, diagnostic accuracy

## Abstract

**Background:** Diabetic retinopathy (DR) is a leading cause of visual impairment worldwide. Manual grading of fundus images is the gold standard in DR screening, although it is time-consuming. Artificial intelligence (AI)-based algorithms offer a faster alternative, though concerns remain about their diagnostic reliability. **Methods:** A cross-sectional pilot study among patients (≥18 years) with diabetes was established for DR and diabetic macular edema (DME) screening at the Oslo University Hospital (OUH), Department of Ophthalmology, and the Norwegian Association of the Blind and Partially Sighted (NABP). The aim of the study was to evaluate the validity (accuracy, sensitivity, specificity) and reliability (inter-rater agreement) of automated AI-based compared to manual consensus (MC) grading of DR and DME, performed by a multidisciplinary team of healthcare professionals. Grading of DR and DME was performed manually and by EyeArt (Eyenuk) software version v2.1.0, based on the International Clinical Disease Severity Scale (ICDR) for DR. Agreement was measured by Quadratic Weighted Kappa (QWK) and Cohen’s Kappa (κ). Sensitivity, specificity, and diagnostic test accuracy (Area Under the Curve (AUC)) were also calculated. **Results:** A total of 128 individuals (247 eyes) (51 women, 77 men) were included, with a median age of 52.5 years. Prevalence of any vs. referable DR (RDR) was 20.2% vs. 11.7%, while sensitivity was 94.0% vs. 89.7%, specificity was 72.6% was 83.0%, and AUC was 83.5% vs. 86.3%, respectively. DME was detected only in one eye by both methods. **Conclusions:** AI-based grading offered high sensitivity and acceptable specificity for detecting DR, showing moderate agreement with manual assessments. Such grading may serve as an effective screening tool to support clinical evaluation, while ongoing training of human graders remains essential to ensure high-quality reference standards for accurate diagnostic accuracy and the development of AI algorithms.

## 1. Introduction

Diabetic retinopathy (DR) is the most common late complication of diabetes mellitus (DM) [1], and the major cause of secondary blindness and reduced vision in people aged 20 to 75 years worldwide [2,3,4,5,6]. In Norway, the prevalence of DR was 28% nationwide (66% for type 1 DM (T1DM) and 24% for type 2 DM (T2DM) in the years 2006–2007), and 58.3% and 23.1%, respectively, in the years 2022–2023 [7,8]. Fundus photography screening appears to be an effective tool for detecting DR [9] and has been proven to be cost-effective in reducing blindness and visual impairment [10,11]. The DR screening program in England and Wales after 10 years from implementation showed a reduced incidence of newly blind due to DM to around 20% [12].

Although in other Nordic countries like Denmark, Sweden, Iceland, and Finland, screening programs and patient registers for DM have existed for many years, in Norway, such screening programs have only been recently implemented in the Oslo region [13,14,15]. Despite the WHO’s guidelines, which recommend annual DR screening of patients with DM, and biennially, in case of well-controlled blood sugar and no signs of DR, the screening rate in Norway is only around 65–70% [16]. This falls short of the goals set by the St. Vincent Declaration in the late 1980s and the Liverpool Declaration in 2005, which aimed to reduce visual impairment from DR by 2010 through systematic screening of at least 80% of people with DM [17,18]. Norway has among the highest prevalences of T1D in the world [19]; thus, more resources would be needed for DR screening of this group of patients.

In 2018, the Norwegian Directorate of Health published national professional guidelines to improve access to DR screening, regulate screening intervals, and standardize imaging and grading for the diagnosing of DR [20]. These guidelines were further detailed in 2022, incorporating recommendations from the International Council of Ophthalmology (ICO) and other Nordic countries [21]. In addition, the use of artificial intelligence (AI) for grading of DR has not yet been defined. The scarcity of ophthalmologists and their uneven distribution in Norway make running a DR screening program a challenge. According to the Norwegian National Health and Hospital Plan 2020–2023, the use of AI in healthcare services is recommended, provided it is beneficial for the patient. Automated grading and other AI-based grading modalities can contribute to more efficient use of healthcare resources with high specificity and sensitivity [10,22,23,24,25,26,27]. Numerous studies have evaluated AI systems’ ability to detect referable DR (RDR), defined as severity level moderate non-proliferative DR (NPDR) and above. These AI performances were compared to the high-standard human grading, assessing their diagnostic accuracy and reliability in identifying cases needing medical attention [26,28,29]. However, systematic reviews have argued that AI applications have variable performance, low specificity, poor methodological quality, and a high risk of bias, which makes it difficult to generalize AI use in real clinical settings; thus, validation is needed [30,31,32]. Countries like the U.S., U.K., Singapore, Australia, India, and China have integrated AI into their national DR screening programs, conducting research to test various AI systems [26,29,33]. All these studies, which tested the performance of different AI systems, found that AI showed sensitivity and specificity of at least 80%, and a majority over 90% in detecting RDR [26,27,29,33], as in a large population study in England, where the AI system achieved a sensitivity of 95.4% and specificity of 92.0% for identifying RDR [34].

Unlike most studies focusing on inter-grader agreement in DR grading either among different healthcare professionals or between single-profession manual graders and autonomous AI, this pilot clinical study aimed to compare the validity (accuracy, sensitivity, specificity) as well as reliability (inter-rater agreement) of AI-based grading as a screening tool for DR and diabetic macular edema (DME), among patients with DM in Oslo, Norway, utilizing both grading of manual consensus (MC), consisting of an ophthalmologist, an optometrist, and an ophthalmic nurse and automated by AI.

## 2. Methods

### 2.1. Study Design, Population, and Fundus Imaging

A cross-sectional, pilot study was conducted in accordance with the Declaration of Helsinki and approved by the Regional Committee for Medical and Health Research Ethics no. 388,111 (14 June 2022) and the Data Protection Officer (DPO) at Oslo University Hospital (OUH), no. 22/11849. The study took place both at the Department of Ophthalmology, OUH, and an external imaging station at the Norwegian Association of the Blind and Partially Sighted (NABP) between October 2022 and November 2023. Adult patients with DM (18 years and above) who were referred mainly by general practitioners and scheduled for screening for DR at OUH were asked to participate. In addition, we wanted to address people with DM, primarily, not followed by an ophthalmologist, and screen them accordingly for DR at NABP. Information about the project and a link to a booking system online for scheduling the appointment were advertised both online (website for OUH and University of Oslo (UIO) employees, the webpage of NABP and webpage of Diabetes Society and their social media pages: Instagram pages, Optometrist site on Facebook, LinkedIn), and also as written information with a QR-code linked to the project site on the NABP webpage, distributed throughout the city of Oslo. Informed consent was obtained from all participants.

The inclusion criteria of the study were diagnosis of DM, age 18 years and older, willingness to participate in a study, no known previously diagnosed eye disease, and availability of a gradable digital retinal image in at least one eye. The exclusion criteria were eyes with ungradable digital retinal images, eyes suffering from an eye disease, such as a nuclear cataract, which could affect image clarity, eyes that have undergone retinal surgery, or eyes with no visual potential. In addition, images taken on the Optos ultra-widefield retinal imaging device were excluded.

The enrolment criteria required no information about the patient’s general or ophthalmic health, including DR diagnosis. However, in addition to information about the age and gender, other information being collected was type and duration of DM, glycosylated hemoglobin (HbA1c), blood pressure (BP), and follow-up time, if known at NABP; or from the patient’s journal at OUH, when available.

Non-mydriatic fundus cameras used in the project were CLARUS TM 700, Zeiss (Carl Zeiss Meditec AG, Jena, Germany), with a 133° field of view at OUH, and iCare DRS Plus TrueColor confocal fundus imaging system (Centervue, Padua, Italy), with a 45° × 40° field of view at NABP. A total of 2 images per eye were taken, by experienced nurses at OUH, and by the same optometrist at NABP, the latter acquiring a disc- and a macula-centered image for each participant. The image quality was evaluated manually by photographers at the site and repeated if it seemed to be insufficient for grading.

The results were, in short, explained to the patients. All study participants at NABP were asked if they wanted to continue follow-up at the regular DR screening program at OUH, and all of them wished to be referred there. Their images were sent together with all collected information and a referral to the OUH screening section for further validation.

### 2.2. Grading of DR and Diabetic Macular Edema

All images were graded according to the International Clinical Disease Severity Scale for DR (ICDR), developed by the International Council of Ophthalmology [35,36,37]. The scale consists of the following: no DR (ICDR0) with the absence of visible retinal changes; mild NPDR (ICDR1), with only microaneurysms (MA) or dot hemorrhages present; moderate NPDR (ICDR2), with more advanced changes than mild NPDR, but less than severe; severe NPDR (ICDR3), with extensive intraretinal hemorrhages, venous beading, or prominent intraretinal microvascular abnormalities (IRMA), adhering to the 4-2-1 rule; proliferative DR (PDR) (ICDR4) with neovascularization, on either vitreous/preretinal hemorrhages or proliferative growth on the optic disc (NVD) or elsewhere (NVE), and DME.

DME manifests as retinal thickening near the macula’s center. When three-dimensional assessment methods, such as stereo fundus photography or OCT, are unavailable (not used in this study), hard exudates within one disc diameter (1DD) from the macular center serve as an indicator for detecting DME. The latter was classified according to Wilkinson et al. severity scale [37], as either clear macula (no diabetic maculopathy), or presence of hard exudates within 1DD from the foveola.

In contrast to the usual screening procedure, when a patient is treated according to the worst DR grade in either eye (patient level), the grading and analysis were performed for each eye separately (eye level), and images were graded independently.

Manual grading of DR was performed by a consensus of healthcare professionals (optometrist, ophthalmic nurses (certified graders), and an experienced ophthalmologist) considered a reference (golden) standard. Images were graded together in the same room, on the same screen, after the patients’ visit had taken place. Thereafter, the images were downloaded into EyeArt (Eyenuk) and analyzed for automatic screening.

### 2.3. Autonomous AI Diagnostic System/Automated DR Grading Software

Fundus images were graded by an autonomous AI-based DR detection system, EyeArt version v2.1.0 (Eyenuk, Inc., Los Angeles, CA, USA), a fully automated, cloud-based software that analyses retinal images and provides screening recommendations. Using advanced algorithms and deep learning, it assesses DR severity based on the ICDR or the U.K.’s NHS Diabetic Eye Screening Programme (NDESP) severity scale and identifies Clinically Significant Macular Edema (CSME) by detecting hard exudates near the macula. EyeArt has both FDA approval for detecting DR in the United States, and CE marking as a class IIb in the European Union (EU) under the EU’s Medical Devices Regulation 2017/745 (“MDR”)—a sole system for the detection of 3 diseases: RDR and vision-threatening DR (VTDR), AMD, and glaucomatous optic nerve damage [38,39]. For each analysis, EyeArt requires 2 × 45-degree color fundus photographs for each eye: macula-centered and optic-nerve-centered). The ultra-widefield (UWF) images from the CLARUS TM 700 camera were cropped so that they could closely fit the 45-degree field of view in order to upload them correctly into the EyeArt software.

Since EyeArt cannot analyze images for just one eye, any patient with an ungradable image in a single eye automatically had both eyes reported as ungradable. To address this issue, it was decided to upload the same images for both eyes, allowing us to obtain results for the one eye with gradable images.

### 2.4. Statistical Analysis

Descriptive statistical analysis was performed and presented in the form of percentages (%), median, interquartile range (IQR), and ranges (minimum and maximum). The normality of continuous variables was tested on a histogram, Q–Q plot, and by the Shapiro–Wilk test. Spearman correlations were used to measure the strength and direction of the association between ordinal variables. Data are presented with Spearman’s correlation coefficient (r) and with Bonferroni-adjusted *p*-values. Spearman’s correlation is categorized as follows: <0.4 (weak), 0.4–0.7 (moderate), and over 0.7 (strong) correlation [40].

The Quadratic Weighted Kappa (QWK) and Cohen’s Kappa (κ) were used to test the strength of overall agreement between MC and AI-based grading of DR in case of ordinal variables (grades) with 4 categories (0: no, 1: mild, 2: moderate, 3: severe DR) and in case of DME two categories (0—No; 1—Yes). The strength of the agreement was assessed using the Landis and Koch approach [41], where 0.20 = poor; 0.21–0.40 = fair; 0.41–0.60 = moderate; 0.61–0.80 = good; and 0.81–1.00 = very good agreement [42]. Sensitivity and specificity of MC vs. AI grading were also calculated and compared for all types of DR and RDR. Data are presented with percentage, *p*-value, and 95% CI.

The Area Under the Curve (AUC) was used to measure the accuracy of a quantitative diagnostic test. It is categorized as follows: 0.90–1.00 (excellent), 0.80–0.90 (good), 0.70–0.80 (fair), 0.60–0.70 (poor), and 0.50–0.60 (fail). Data are presented with the AUC and with their 95% CI. All statistical analyses were based on paired eye-level comparisons between the MC and AI.

Mean imputation was performed in case the continuous variables (age, HbA1c, systolic, diastolic blood pressure (SBP/DBP)) were normally distributed, had no outliers, and missing values were missing at random (MAR).

All differences were considered significant at *p* < 0.05. Statistical Package for STATA (Stata version 17.0 SE-Standard Edition; College Station, TX, USA) was used for the statistical analysis.

## 3. Results

**Figure 1:**
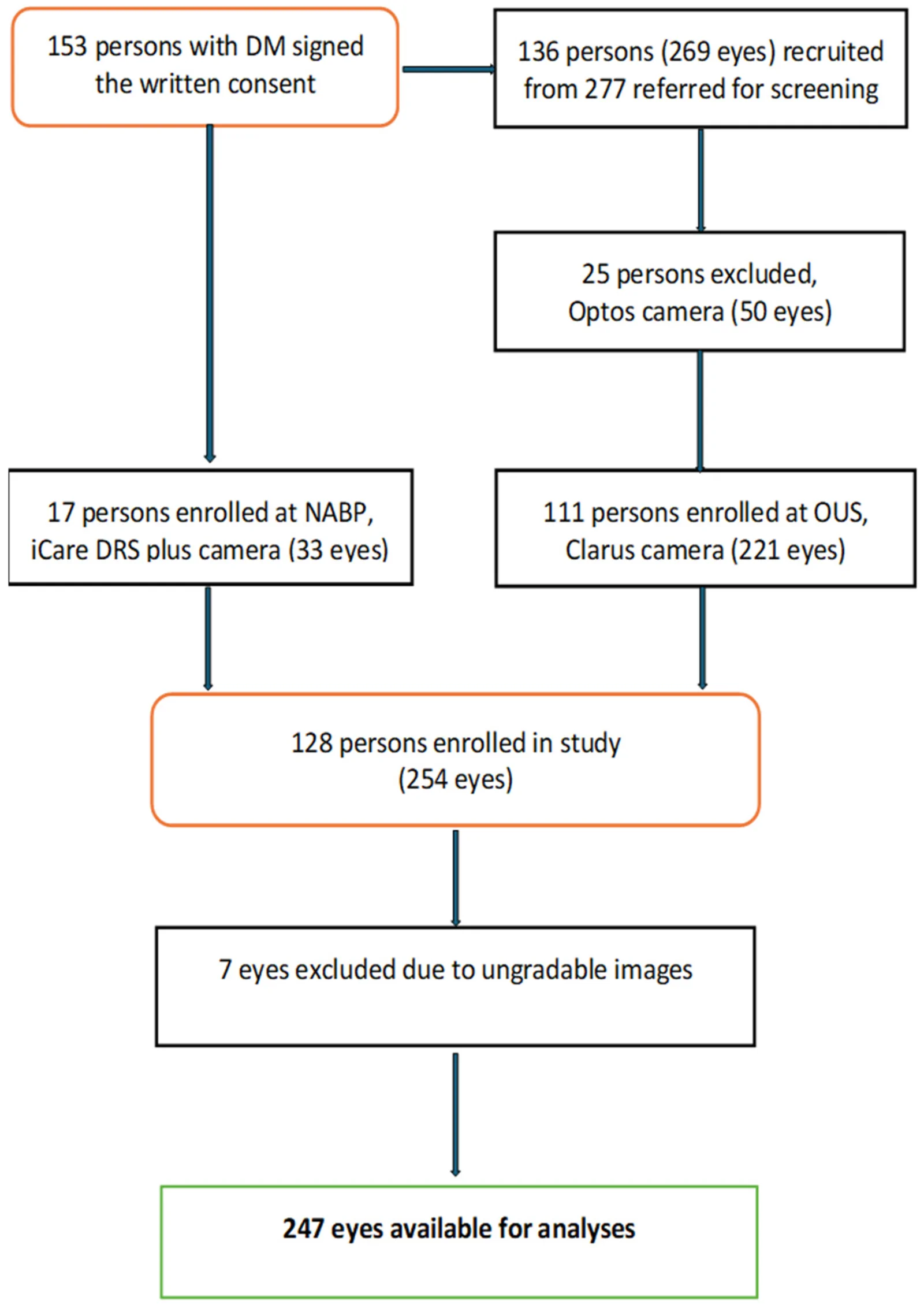
Flow chart of the study population, as well as the inclusion and exclusion criteria.

A total of 153 patients with DM from the city of Oslo (Southeast Health region) signed the written consent to participate in the study. Among them, 17 persons (33 eyes) were screened for the first time at NABP, while 136 persons (269 eyes) were screened at OUH. Twenty-five participants from OUH were excluded since images were taken on an Optos widefield camera and could not be analyzed by EyeArt. Of the 277 screenings conducted during this period, 111 participants (214 eyes) from OUH comprised both new and follow-up cases. One of the remaining 111 (221 eyes) participants was treated with laser in one eye, and that eye was excluded from the analysis. Among the study participants from NABP, one had lost vision in 1 eye, so 33 eyes were included. Images from 128 participants (254 eyes) were included for grading. Seven eyes were excluded due to poor image quality or missing data—two persons had images ungradable by both graders—EyeArt and MC; three were ungradable by only human graders; and two only by EyeArt. A total of 128 persons (247 eyes) were eligible for the final analysis (Figure 1).

Table 1 presents the characteristics of the study participants. Altogether 128 participants—51 women (39.8%) and 77 men (60.1%)—were included in the analysis. The median age of the study participants was 52.5 years (IQR: 44.5–64.5, range: 18–89 years). A total of 31 (24.2%) participants had T1D, including 2 with Latent Autoimmune Diabetes in Adults (LADA), and 97 (75.8%) had T2D, including 1 with Maturity-Onset Diabetes of the Young (MODY3). The median duration of DM was 4.5 years (IQR: 1.0–8.0, range: 0.1–42.3). The median HbA1c was 55.5 mmol/mol (IQR: 48.0–60.0, range: 31.0–125.0). The median SBP and DBP were 130 mmHg (IQR: 122.0–140.0; range: 90.0–164.0) and 79.8 mmHg (IQR: 79.4–80.0, range: 60.0–100.0), respectively.

**Figure 2:**
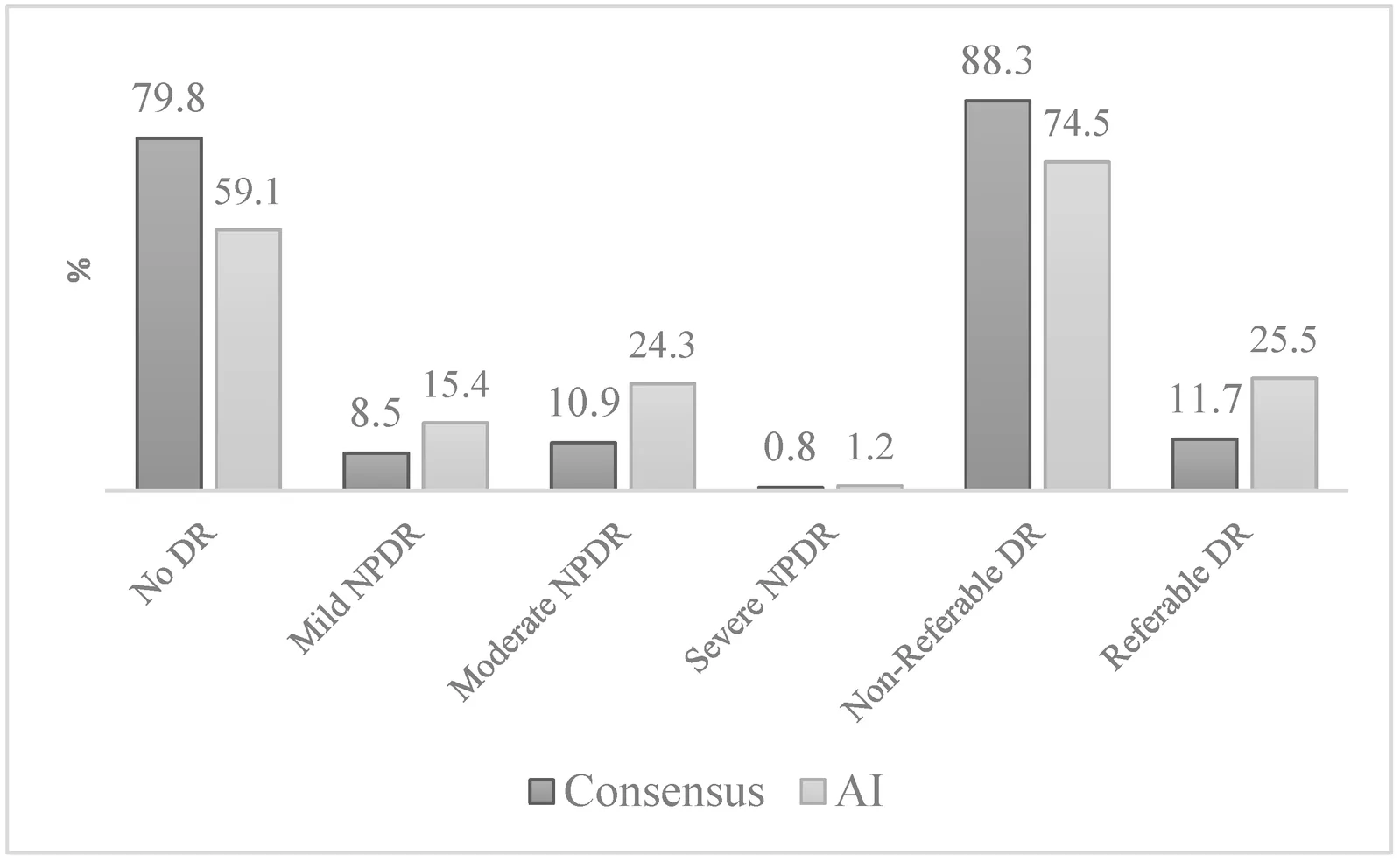
Percent distribution of the stages of diabetic retinopathy according to the grading of manual consensus and AI-based EyeArt method. (DR: diabetic retinopathy; NPDR: non-proliferative DR; AI: artificial intelligence).

The distribution of DR by the MC vs. AI is shown in Figure 2. The grading for any level of DR by the MC and AI were no (79.8% vs. 59.1%), mild (8.5% vs. 15.4%), moderate (10.9% vs. 24.3%), and severe NPDR (0.8% vs. 1.2%), respectively. None of the study participants had PDR. The distribution of non-RDR vs. RDR was (88.3%, 74.5%) vs. (11.7%, 25.5%), respectively. DME was present in one eye (0.4%).

Table 2 presents the distribution of DR grading by the AI compared to the MC grading: 143 eyes were classified as no, 13 as mild, 22 as moderate, and 1 participant as severe DR. Any DR was identified in 50 eyes by AI and in 101 eyes by MC grading.

Table 3 presents the overall agreement, sensitivity, specificity, AUC, and prevalence for detecting any type of DR and RDR using AI grading vs. MC. A significant moderate agreement and correlation were observed between the two grading methods for detecting any type of DR (QWK: 0.52; 95% CI: 0.50–0.58; Spearman’s r: 0.56) and RDR (QWK: 0.48, 95% CI: 0.35–0.61; Spearman’s r: 0.54) (*p* < 0.001).

The sensitivity for detecting any type of DR was 94.0% (95% CI: 91.0–96.9), while for RDR, it was 89.7% (95% CI: 85.9–93.4). Specificity was 72.6% (95% CI: 67.0–78.1) for any type of DR and 83.0% (95% CI: 78.5–87.7) for RDR.

The AUC demonstrated good diagnostic performance, with 83.5% (95% CI: 78.3–88.7) for any type of DR and 86.3% (95% CI: 79.3–93.4) for RDR.

The prevalence of any type of DR was 20.2% (95% CI: 15.2–25.2), while for RDR, it was 11.7% (95% CI: 7.7–15.8), respectively.

## 4. Discussion

This cross-sectional pilot study aimed to evaluate the validity and reliability of automated AI grading of DR compared to MC grading performed by a multidisciplinary team of different healthcare professionals. Our findings reveal that the AI system demonstrated promising performance characteristics, highlighting its potential as a viable screening tool for DR.

The AI system achieved a sensitivity of 94% for any DR and 89.7% for RDR, exceeding the British Diabetic Association’s (BDA) requirement of 80%. However, its specificity was below the BDA’s 95% criterion, with 72.6% for any DR, and 83.0% for RDR. These results underscore the need to refine AI algorithms to reduce false positives and enhance alignment with established diagnostic standards [43,44,45].

EyeArt typically exhibits high sensitivity and strong capability in detecting eye pathology, especially in advanced stages, as evidenced by numerous studies [22,46,47]. While our previous research showed 100% sensitivity and specificity using the same AI system in a cohort of minority women [47], challenges remain, as reports indicate variability in specificity, with some studies noting lower specificity compared to human grading [48,49,50,51]. Our study’s findings are consistent with the observed lower specificity in a screening of 260 patients for RDR using EyeArt software compared to optometrist grading, with 100% sensitivity but only 77.8% specificity. The authors suggest that laser scars, drusen, or artifacts detectable by AI—but not by human eyes—could be responsible for the reduced specificity [51]; however, such changes were not present in the images of our cohort. The lower specificity was also found in a U.K. study involving 30,405 retinal images, where EyeArt showed a sensitivity of 95.7% but the specificity was only 54.0% [50].

The lower specificity of the AI grading system in our study can be attributed to the fact that the Zeiss CLARUS TM 700 camera had not yet been validated for use with the EyeArt software at the time of the study. The FDA requires a specific fundus camera model for the software, while Europe allows the use of any fundus camera, showing the differing regulatory approaches between the USA and Europe [38]. While the CLARUS camera system produces UWF, high-resolution images, the DRS Plus system provides narrower-field images. This difference in image quality and field of view could impact the consistency between AI and human grading.

The distribution of severity level of DR grading by the MC and AI also shows some disparities, with a 1.35 times higher prevalence of cases classified as “No-DR” by the MC grading compared to the AI. The latter identified DR in 50 eyes, while the MC grading detected it in 101 eyes, showing agreement in 178 out of 247 eyes (72.1%).

Conversely, AI tends to classify more cases as mild (15.4%) or moderate (24.3%) NPDR compared to the MC (8.5% and 10.9%, respectively). Such a moderate level of agreement was found for both any DR and RDR. This discrepancy aligns with findings from a study by Heydon et al., which reported AI specificity for “No-DR” at 68% [50]. Similar patterns have been observed in other studies, both in Europe, particularly utilizing true-color, widefield confocal scanning images [52], and in Asia [53]. This could suggest that AI might be more sensitive to early DR indicators, detecting features that may be overlooked by human graders, or capturing more borderline cases, potentially reducing false negatives. While this could minimize the risk of undetected morbidity, it also poses the risk of overestimating disease severity, resulting in false positives and unnecessary follow-up. On the other hand, it has been demonstrated that human graders misclassify DR in 21.6% of cases, particularly at lower stages as “No-DR” and mild NPDR [54]. The grading discrepancies in DR have been attributed to the identification of non-DR retinal lesions, such as RPE atrophy and hypertrophy, retinal telangiectatic vessels, and retinal vein occlusion, as well as the misinterpretation of the internal limiting membrane (ILM) appearing as shiny dots mistaken for exudates [55,56]. In the current study, the grading results might be influenced by image quality issues as well. Several participants had non-mydriatic images that were blurred, contained reflections, or had shadows, primarily in the periphery. The second eye image, typically the left eye, was blurred due to pupil constriction from the flash used on the right eye. Consequently, seven images were excluded due to poor quality. Although the photographers, for most participants in the study, were experienced nurses at OUH, none were certified DR photographers.

Both MC and AI grading identified very few individuals with severe NPDR, with AI classifying three individuals and MC identifying two. No cases of PDR were detected in either method. This result is also likely indicative of the study cohort being comprised of newly referred patients for screening, as it was found by another recently published study from our clinic [7].

Notably, according to the MC grading, the prevalence of any type of DR in our cohort was 20.2%, which is slightly lower than previously reported [8,57]. Significant regional variations were also observed, especially in T1DM, with the highest prevalence observed in North Norway (78% during 2007–2008), compared to 58.3% in Oslo during 2022–2023, and the islands’ areas of the Norwegian West coast (48% during 2009–2011). Conversely, T2D exhibited minimal fluctuations, with rates consistently between 23% and 25% [7,8,57,58]. The result from our study reflects the predominance of T2D cases in our cohort (three-quarters of the participants) with a well-regulated HbA1c and BP, despite the incomplete records for these risk factors for developing DR. The prevalence of RDR was 11.7%, which is in line with previously reported findings [7].

However, the prevalence of DME, 0.4% (only detected in one study participant), was lower than a recent study from our research group (20.2%) based on fundus images, from which 6.6% confirmed using Optical Coherence Tomography (OCT) [11]. This discrepancy may arise from the use of different grading scales. Specifically, the EyeArt AI grading system employs the Wilkinson et al. scale [37], which identifies DME exclusively based on the presence of exudates in the macula. Similarly, in our study, MC grading also recognized DME solely on the existence of exudates. In contrast, the comparative study included both MA and exudates as markers of diabetic maculopathy/DME. Our results are more congruent with findings from the Tromsø Eye Study, which used the same grading scale and noted a DME prevalence of 3.9% [57]. Additionally, the lower prevalence in our study likely reflects the status of participants as being newly referred for screening.

The strength of our study is that it is the first to compare the grading of MC of different healthcare professionals, and the first real-world use of an AI grading system in a cohort in Norway, building upon a previous pilot study performed by us on a cohort of minority women in Oslo [47]. The current study included a diverse group of randomly selected individuals with DM from the Oslo region, representing various ethnic backgrounds. We employed well-defined statistical methods, enhancing the accuracy and reliability of our analysis.

This study also has some limitations. The sample size is too low to generalize the results to the entire population. A further limitation is the heterogeneity of the cohort. Participants were recruited from two distinct sources: newly screened individuals at a community screening site (NABP), and patients referred to, or already under follow-up at a tertiary hospital (OUH). This resulted in a mix of first-time screeners and individuals with varying durations of DM and prior eye care exposure. Additionally, the cohort included both T1D and T2D patients, and incomplete clinical data were available for a subset. A significant number of study participants had missing information on risk factors from the general practitioners or lacked knowledge about HbA1c, duration of DM, and BP. These factors may influence the observed prevalence of DR and limit the generalizability of prevalence estimates to the broader population with DM in Oslo. While this heterogeneity does not affect the primary objective—comparing AI and MC grading—it should be considered when interpreting the prevalence results. Moreover, this is a single-center study that included in the MC group one professional from each affiliated group of ophthalmologists, optometrists, and ophthalmic nurses. The MC grading used as the reference/golden standard assumes superiority over individual expert grading but lacks direct comparative evidence, and the absence of a certified reading center may limit the reliability of our findings. While the MC approach aims to minimize variability among graders, some inter-grader variability may still exist, underscoring the need for cautious interpretation of the results and further investigation into grading consistency in future studies. Additionally, we conducted an eye-level analysis rather than a patient-level analysis, assessing each eye independently, which may affect the evaluation of prevalence and risk factors. To derive AI results, when images from one eye were ungradable, images from the “good” eye were duplicated and entered into the AI grading system. EyeNuk confirms that this approach does not negatively impact the results.

A notable limitation observed in our results was the significant discrepancy in 32 eyes where AI graded the images as “Moderate DR,” whereas the MC assessment indicated “No DR.” This represents a two-step grading difference, which is clinically relevant and could lead to overreferral/overestimation of patients needing follow-up or treatment. These discrepancies reduce the sensitivity of the AI system and raise concerns regarding its reliability in distinguishing between RDR and no DR.

Despite these concerns, systematic reviews suggest AI can effectively identify DR in diverse settings, highlighting its potential to help alleviate the healthcare burden in both high-income and low- and middle-income countries [25,27].

In 2021, a comprehensive evaluation of various AI algorithms showed significant variability in performance, influenced by DR prevalence, mydriasis, and ethnic diversity. This underscores the importance of external validation to ensure algorithms’ efficacy across different populations and clinical settings [32].

Although numerous studies have examined inter-grader agreement between various grading modalities—both human and AI-based systems—the diversity of grading scales for DR and different reference standards complicates comparisons. Moreover, the use of different cameras, the number of retinal fields graded, and differences in whether pharmacological pupil dilation/mydriasis is employed before photography, all exacerbate the difficulty in drawing consistent conclusions across these studies [25,31]. That highlights the importance of having standardized grading systems and screening recommendations. While previous research demonstrated that AI systems can quickly and precisely analyze large volumes of images, offering cost-effective solutions and reducing the burden on healthcare professionals, the current study reveals that such systems require refinement to improve agreement with human graders [47]. Although AI grading generally corresponded with MC grading, it occasionally overlooked relevant cases, resulting in false negatives, thus requiring cautious interpretation. AI appears to be greatly affected by minor variations between training and testing data sets, which can potentially diminish its effectiveness after implementation. Therefore, further research on AI algorithms is vital to help clinicians select suitable models for clinical use, focusing on assessing performance within the specific populations where they will be implemented [32,59].

Nonetheless, the AI demonstrated the potential of being a good triage tool for identifying patients at risk of moderate to severe DR, who would benefit from further evaluation by specialists. Task-sharing with AI can enhance the screening capacity in DR by shifting from direct ophthalmologist exams to remote methods like retinal photography, tele-screening, and AI-based grading. Advances in screening technologies, including the creation of reading centers, automated image analysis, and tele-ophthalmology, promise to further reduce the need for in-person office visits. Improving access to accurate diabetic eye examinations can enhance adherence to recommended screenings and allow for prompt referral of patients with vision-threatening DR.

## 5. Conclusions

This pilot study conducted in Oslo, Norway, highlights the potential of automated AI grading systems as a supplemental tool for DR screening. AI-based grading showed high sensitivity and acceptable specificity for detecting any DR, though inter-rater agreement for severity grading was moderate and requires further optimization for clinical implementation. Ongoing training for healthcare professionals is crucial to ensure quality in assessments. Ultimately, while AI can assist in early detection and timely referrals, it should complement, rather than replace, human judgment. Future research is needed to validate these findings in larger and more diverse populations.

## Figures and Tables

**Table 1:** Characteristics of the study participants.

	*n* = 128 (%)
**Gender (women/men)**	51/77 (39.8/60.1)
**Age (years)** **Median (IQR) range**	52.5 (44.5–64.5) 18–89
**Type of DM (T1D/T2D)**	31/97 (24.2/75.8)
**Duration of DM (years)** **Median (IQR) range**	4.5 (1.0–8.0) 0.1–42.3
**HbA1c (mmol/mol)** **Median (IQR) range**	55.5 (48.0–60.0) 31.0–125.0
**Systolic BP** (mmHg) Median (IQR) range	130 (122.0–140) 90.0–164.0
**Diastolic BP** (mmHg) Median (IQR) range	79.8 (79.4–80.0) 60.0–100.0

T1D/T2D: type 1 and 2 diabetes; DM: diabetes mellitus; HbA1c: glycosylated; BP: blood pressure; IQR: interquartile range; SD: Standard Deviation; *n*: number; %: percentage.

**Table 2:** Distribution of diabetic retinopathy grading by manual consensus and AI according to the severity level at eye level.

MC	AI				
	***n*** ** = 247 Eyes (%)**	**No DR** ***n* (%)**	**Mild DR** ***n* (%)**	**Moderate DR** ***n* (%)**	**Severe DR** ***n* (%)**
	**No DR**	**143 (72.6)**	22 (11.2)	32 (16.2)	**−**
	**Mild DR**	3 (14.3)	**13 (61.9)**	5 (23.8)	**−**
	**Moderate DR**	**−**	3 (11.1)	**22 (81.5)**	2 (7.4)
	**Severe DR**	**−**	**−**	1 (50.0)	**1 (50.0)**

MC: manual consensus; *n*: number; DR: diabetic retinopathy; AI: artificial intelligence; numbers in bold indicate the highest agreement between AI and manual consensus.

**Table 3:** Level of agreement, sensitivity, specificity, diagnostic test accuracy, and prevalence of AI vs. manual consensus for detecting diabetic retinopathy.

AI vs. MC	Any Type of DR *n* = 247	RDR *n* = 247
**QWK** (95% CI) Spearman’s r	0.52 (0.50–0.58) 0.56	0.48 (0.35–0–61) 0.54
**Sensitivity** (%, 95% CI)	94.0 (91.0–96.9)	89.7 (85.9–93.4)
**Specificity** (%, 95% CI)	72.6 (67.0–78.1)	83.0 (78.5–87.7)
**AUC** (%, 95% CI)	83.5 (78.3–88.7)	86.3 (79.3–93.4)
**Prevalence** (%, 95% CI)	20.2 (15.2–25.2)	11.7 (7.7–15.8)

MC: manual consensus; DR: diabetic retinopathy; RDR: referable diabetic retinopathy; QWK: Quadratic Weighted Kappa; *n*: number; AI: artificial intelligence; CI: Confidence Interval; *p* < 0.05. The strength of the agreement (QWK): 0.20 = poor; 0.21–0.40 = fair; 0.41–0.60 = moderate; 0.61–0.80 = good; and 0.81–1.00 = very good agreement. Spearman’s r is categorized as follows: <0.4: weak, 0.4–0.7: moderate, >0.7: strong correlation. (AUC) Area Under the Curve: expressed as percentages: 90–100% (excellent), 80–89.9% (good), 70–79.9% (fair), 60–69.9% (poor), and 50–59.9% (fail).

## Data Availability

The data presented in this study are available on request from the corresponding author.
